# Effects of cereal breakfasts on postprandial glucose, appetite regulation and voluntary energy intake at a subsequent standardized lunch; focusing on rye products

**DOI:** 10.1186/1475-2891-10-7

**Published:** 2011-01-19

**Authors:** Liza AH Rosén, Elin M Östman, Inger ME Björck

**Affiliations:** 1 From the division of Applied Nutrition and Food Chemistry, Department of Food Technology, Engineering and Nutrition, Lund University, P.O. Box 124, SE-221 00, Sweden

## Abstract

**Background:**

Rye products have been demonstrated to lower the acute insulin demand, induce a low and prolonged blood glucose response (high Glycemic Profile, GP) and reduce subclinical inflammation. These products may therefore contribute to a lowered risk of obesity, type 2 diabetes and cardio vascular disease. The objective of the present paper was to evaluate the mechanism for a reduced postprandial insulin demand with rye products, and to explore possible appetite regulating properties.

**Methods:**

10 healthy subjects were served breakfast meals (50 g of available starch) with endosperm- or whole grain rye breads, with and without lactic acid, boiled whole grain rye- (RK) or wheat (WK) kernels, or white wheat bread reference (WWB) in random order in a cross-over design. Plasma concentrations of glucose, ghrelin, serum insulin, free fatty acids, adiponectin, breath hydrogen excretion (H_2_), and subjective satiety was evaluated during the postprandial phase. 270 min after the breakfast, an ad lib lunch buffet was served and the voluntary energy intake (EI) was registered.

**Results:**

All rye products and WK induced lower insulinemic indices (II) than WWB. A lower incremental insulin peak following breakfast correlated with a lower EI at lunch (r = 0.38). A low II was related to improved satiety in the early postprandial phase (fullness AUC 0-60 min, r = -0.36). RK induced a higher GP compared to WWB and WK. A higher GP was related to a lowered *desire to eat *before lunch (AUC 210-270) and to a lower concentration of ghrelin in the late postprandial phase after breakfast (270 min), r = -0.29 and -0.29), which in turn was related to a lower voluntary EI (r = 0.43 and 0.33). The RK breakfast improved satiety in the early postprandial phase (0-60 min) compared to WWB, and induced a lower EI at lunch (-16%). A high content of indigestible carbohydrates in the breakfast products was related to improved satiety (0-60 min, r = 0.68 for fullness), and a higher breath H_2 _in the late postprandial phase (120-270 and 270-390 min, r = 0.46 and 0.70). High H_2 _(AUC 120-270 min) also correlated with lower EI (r = -0.34).

**Conclusions:**

Rye products, rich in indigestible carbohydrates, induce colonic fermentation already post the breakfast meal, and lowers acute insulin responses. A high excretion of breath H2 also correlated with a higher GP. Especially, rye kernels induced a high GP which was associated with a 16% lowering of energy intake at a subsequent lunch meal. The bulking effect of rye fiber, colonically derived fermentation metabolites, a high GP and a low insulin response possibly all contributes to the benefits on glucose- and appetite regulation seen in an acute and semi-acute perspective.

## Background

The prevalence of obesity and type 2 diabetes (T2D) is increasing globally and preventive strategies are urgently needed. Whole grain foods have been shown to facilitate weight regulation and to lower the risk of T2D, cardio vascular disease (CVD) and the metabolic syndrome [1-8]. The protective mechanism may be related to the presence of dietary fiber or other potentially bioactive components [9,10]. In addition, some whole grain products are characterized by a low glycemia and insulinemia, properties also known to protect against T2D and CVD [3,11-14]. Products made from rye (*Secale cereale*) have previously been shown to induce a low insulin response [15-18] which may counteract development of insulin resistance [19]. Furthermore, evidence of a facilitated glycemic regulation has been found with rye products in healthy subjects, with lower incremental peaks, avoidance of hypoglycemia, and glucose excursions remaining above fasting for a longer time i.e. a higher glycemic profile (GP) [18]. The mechanisms behind the beneficial glycemic and insulinemic responses of rye products are, however, not known.

Avoidance of frequent and elevated blood glucose excursions is protective against oxidative stress and subclinical inflammation [20]. Furthermore, several studies have shown that carbohydrate foods causing low postprandial insulin responses induce higher satiety and a lower voluntary food intake at a subsequent meal, as compared with foods inducing high acute insulinemia [21-25]. In light of the low acute insulin responses commonly induced by rye products, such products could be anticipated to possess appetite regulating properties. In support of this, meal studies with rye products in healthy adults demonstrated that a low insulin response was associated with less accentuated late postprandial hypoglycemia, lowered sense of hunger, and lowered late postprandial increase of plasma ghrelin [18]. These findings indicate that further insights regarding the metabolic- and appetite regulating properties of rye products might add to the knowledge regarding mechanisms for health benefits of whole grains.

Since rye bread products are frequently processed using sour-dough fermentation, the potential effect of organic acids also deserve attention in this context. Lactic acid addition to bread has previously been reported to lower glycemic response [26].

In the present study, the metabolic and appetite regulating properties of rye breads made from endosperm or wholegrain rye flour, were investigated in healthy subjects, using white wheat bread as reference (WWB). The effect of adding lactic acid to the rye breads was also studied. In addition, test products made by boiling of kernels from rye and wheat, respectively, were included. The test products were provided as breakfasts and postprandial plasma were analyzed for measures of glucose metabolism (glucose, serum insulin, FFA, adiponectin), and appetite regulating hormones (ghrelin). Additionally markers of colonic fermentation were measured (breath hydrogen excretion). Finally, subjective satiety was evaluated in the postprandial phase. At 270 min after the breakfast, an *ad libitum *buffet lunch meal was served and the energy intake was registered.

## Methods

### Test subjects

Ten healthy non-smoking volunteers (5 men and 5 women) aged 26.0 ± 1.1 y with normal body mass indices (22.6 ± 0.4 kg/m^2^) and without drug therapy participated in the study. All subjects had normal fasting plasma glucose concentrations (5.5 ± 0.1 mmol/L). The subjects were recruited in March 2007 and the study was performed from April to June 2007. All subjects gave their informed consent and were aware of the possibility of withdrawing from the study at any time they desired. Approval of the study was obtained from the regional ethical review board in Lund, Sweden (reference number 109/2007)

### Breakfast products

Four rye breads, boiled rye and wheat kernels and a white wheat bread reference were included in the study. Whole grain rye flour, kernels and endosperm rye flour (commercial blends) were provided by Lantmännen R&D (Järna, Sweden). Commercial white wheat flour was obtained from Kungsörnen AB (Järna, Sweden). Whole wheat kernels (Tiger) were provided by BFEL (Germany). Dry yeast was obtained from Jästbolaget AB (Sollentuna, Sweden) and lactic acid (88-92% extra pur) from Riedel-de Haën (Morris Township, NJ, USA).

#### WWB

The white wheat bread (WWB) was made from 540 g of white wheat flour, 360 g water, 4.8 g dry yeast, 4.8 g NaCl and baked in a bread baking machine (BM 3983, Severin, Sundern, Germany) using a program for white bread.

#### ERB

Endosperm rye bread (ERB) was made from 5000 g endosperm rye flour, 3413 g water, 84 g dry yeast and 43 g NaCl (containing 5 mg KI/100 g). The dough was mixed for 8 minutes and proofed at room temperature for 30 minutes. It was divided into pieces of 1000 g each and placed in baking tins. The dough was subjected to a second proofing (38°C, 85 % humidity) during 30 minutes for the endosperm rye breads and 45 minutes for the whole grain rye breads. Baking was performed initially at 250°C with 3 sec of steam. The temperature was then immediately lowered to 200°C and the breads were baked for 40 min.

#### ERB-lac

Endosperm rye bread with lactic acid (ERB-lac) was made from 5000 g endosperm rye flour, 3322 g water, 90 g lactic acid, 84 g dry yeast and 43 g NaCl (containing 5 mg KI/100 g). The bread was made using the same method as for ERB.

#### WGRB

Whole grain rye bread (WGRB) was made from 5000 g coarse whole grain rye flour, 3661 g water, 84 g dry yeast and 43 g NaCl (containing 5 mg KI/100 g). The bread was made using the same method as for ERB but was baked for 45 min.

#### WGRB-lac

Whole grain rye bread with lactic acid (WGRB-lac) was made from 5000 g coarse whole grain rye flour rye flour, 3574 g water, 90 g lactic acid, 84 g dry yeast and 43 g NaCl (containing 5 mg KI/100 g). The bread was made using the same method as for WGRB.

The WWB was left to cool for 1 hour and the rye breads for 21 hours under cover. Thereafter, the crust was removed and the breads were sliced and wrapped in aluminum foil in portions sizes, put into plastic bags and stored in a freezer (-20°C) until use. The day before the experiment, the breads were taken from the freezer and were thawed over night at ambient temperature, still wrapped in aluminum foil and in the plastic bag.

#### RK and WK

The wholegrain wheat kernels (WK) and rye kernels (RK) were prepared on the day of the experiment. 97.1 g of whole wheat kernels and 0.5 g NaCl were boiled in 156.4 g water for 40 minutes. 106.6 g whole rye kernels and 0.5 g NaCl were boiled in 189.5 g water for 35 minutes. All water was absorbed by the kernels.

### Composition of the lunch buffet

An *ad libitum *lunch buffet was served at 270 min after breakfast to measure voluntary food intake. The buffet meal was a common Swedish lunch meal and was composed of meatballs (ICA Handlarnas AB, Solna, Sweden), pasta (Kungsörnen AB, Järna, Sweden), ketchup (Procordia Food AB, Eslöv, Sweden) and cucumbers. The cucumbers were fresh, and were peeled and sliced prior to serving to ensure homogeneity. Meatballs (240 g) were heated in a microwave oven (MS 2334B, LG, LG Electronics Inc., Seoul, Korea) for 4 min at 850 W. The pasta (325 g dry weight) was boiled for 8 min in 3 liters of water with 2 teaspoons (13 g) NaCl. One tablespoon of rapeseed oil (Di Luca & Di Luca AB, Stockholm, Sweden) was added to the pasta after boiling.

### Chemical analysis of the breakfast products

Prior to analysis of the total starch, fiber and protein content, the breakfast products were air dried and milled (1.0 mm screen, Cyclotec, Tecator, Höganäs, Sweden). Measurements of resistant starch (RS) was performed on products as is. Total and resistant starch was analyzed according to Björck et al. [27] and Åkerberg et al. [28]. The available starch was calculated by subtracting RS from total starch. Insoluble and soluble dietary fiber were determined with a gravimetric, enzymatic method described by Asp et al. [29]. Protein content was determined by Kjeldahl analysis (Kjeltec Auto 1030 Analyser, Tecator, Höganäs, Sweden). Fat content in the products was calculated using data from endosperm and wholegrain rye and wheat flours from Lantmännen. Energy content of the test meals were calculated using fat, carbohydrate and protein contents of the meals (17 kJ per gram of protein and available carbohydrate and 37 kJ per gram fat). The rate of starch hydrolysis (HI) was determined using an in vitro procedure based on chewing [30], with WWB as a reference. The nutritional composition and HI of the products are shown in Table 1.

**Table 1:** Composition of the breakfast products

Meals	Portion size	Available starch	Total starch	Resistant starch	Protein	Soluble fibers	Indigestible carbohydrates	Water content	Energy content	HI
	***g/portion***								***kJ***	***%***
WWB	124.0	50.0	51.1	1.1	7.3	0.5	4.4	59.0	1 015	100 ± 0 ^a^
ERB	134.8	50.0	51.4	1.4	6.1	4.0	11.9	64.3	1 033	87 ± 3 ^b^
ERB-lac	133.1	50.0	51.7	1.7	6.0	4.5	11.8	62.0	1 033	85 ± 2 ^b^
WGRB	163.4	50.0	52.5	2.5	8.3	4.0	19.8	79.7	1 078	87 ± 2 ^b^
WGRB-lac	158.4	50.0	51.8	1.8	7.9	4.1	17.7	76.7	1 069	84 ± 2 ^b^
RK	226.6	48.4	55.9	7.5	9.2	3.7	25.2	131.9	1 066	70 ± 5 ^c^
WK	171.9	50.3	57.8	7.5	12.2	1.5	19.8	84.4	1 170	72 ± 3 ^c^

Indigestible carbohydrate content consists of soluble fibers, insoluble fibers and resistant starch in the breakfast products. n = 2 (total starch and proteins), n = 3 (fiber content), n = 6 (RK, n = 4, resistant starch), n = 6 (HI). Products not sharing the same letter are significantly different, p < 0.05 (ANOVA, followed by Tukey's test).

### Study design

The test and reference products were provided as breakfasts, on 7 different occasions, in random order separated by approximately 1 wk. The subjects were instructed to eat a standardized evening meal (9:00-10:00 P.M) prior to the test, consisting of a few slices of white wheat bread. No eating or drinking except for small amounts of water was then allowed until the start of the test. The subjects were also told to avoid alcohol and excessive physical exercise the day before each test, and otherwise as far possible maintain their regular life style throughout the entire study. The subjects arrived at the laboratory at 07.45 a.m. on the test day. A peripheral venous catheter (BD Venflon, Becton Dickinson, Helsingborg, Sweden) was inserted into an antecubital vein.

Fasting blood samples were taken prior to the breakfast meal at time 0. Thereafter the test meals, contributing with 50 g of available starch, were served with 250 ml of tap water. The test subjects finished the breads within 14 minutes and the kernels within 25 minutes. At 120 min after the breakfast, the test subjects were served 250 ml of tea, coffee or water without any sweeteners or milk products. The chosen beverage remained consistent for each individual at all 7 visits.

At 270 min after commencing the breakfast meals, the subjects were provided the lunch buffet and were instructed to eat the amount needed to reach comfortable satiation. At the following visits they should eat until they reached the same degree of satiation as on their first occasion. On their first visit, the subjects could drink as much water as they desired, and the same amount of water was then served at the following 6 visits. The subjects had to finish their lunch within 30 min, before the next blood sampling occasion at 300 min after commencing breakfast. The weight of the different food items ingested was registered individually to allow calculation of the energy intake at the buffet lunch meal. The energy content of the foods in the lunch buffet was obtained from the manufacturer of the products, and that of the cucumber from food tables (Swedish National Food Administration).

### Physiological parameters

Capillary blood samples were taken for analysis of plasma glucose (p-glucose). Venous blood samples were drawn for the analysis of serum insulin, serum free fatty acids (s-FFA), s-adiponectin and p-ghrelin. Breath hydrogen excretion (H_2_) was measured as a marker of colonic fermentation, using an EC 60 gastrolyzer (Bedfont EC60 Gatrolyzer, Rochester, England). In addition, the subjects were asked to fill in their subjective *feeling of fullness, hunger *and *desire to eat*, respectively, using a 100 mm Visual Analogue Scale (VAS).

Glucose and insulin were measured at 0, 15, 30, 45, 60, 90, 120, 180, 240 and 270 min. FFA and adiponectin were measured at 0, 180 and 270 min. Ghrelin was measured at 0, 60, 90, 120, 270, 330, 360 and 390 min. Subjective appetite ratings were performed every 30 min throughout the experimental day and also at 15 and 315 min. H_2 _was measured every 30 min.

After sampling, serum and plasma (EDTA) tubes were left in ice bath for approximately 1 h before being centrifuged for 11 min (1800 * g, 4°C,). Serum and plasma were thereafter immediately separated and the samples were frozen at -20°C (serum) or -40°C (plasma) until analysis. Plasma for ghrelin analysis was sampled into tubes containing 500 KIU aprotinin (Bayer HealthCare AG, Leverkusen, Germany) per ml of whole blood.

Glucose was analyzed using a p-glucose analyzer (Glucose 201+, Hemocue, Ängelholm). The s-insulin analysis was performed on an integrated immunoassay analyzer (CODA Open Microplate System; Bio-rad Laboratories, Hercules, CA, USA) using an enzyme immunoassay kit (Mercodia AB, Uppsala, Sweden). S-FFA were analyzed using an enzymatic colometric method (NEFA C, ACS-ACOD method, WAKO CHEMICALS gmBH, Germany). S-adiponectin was analyzed using an enzyme immunoassay kit (Mercodia AB, Uppsala, Sweden), and p-ghrelin with a radioimmunoassay kit (Linco research Inc., St. Charles, MO, USA).

### Calculations and statistical methods

One subject was excluded from the analysis of data from the WGRB breakfast due to having a cold at that particular test day. The data for WGRB is therefore analyzed with n = 9. Data are expressed as means ± SEM.

The total area under curve (AUC) was calculated for each subject and test meal, using the trapezoid model. The glycemic index (GI) is defined as the incremental positive area under the blood glucose curve after a test product, expressed as a percentage of the corresponding area after an equi-carbohydrate reference product taken by the same subject [31]. The insulinemic index (II) is calculated from the corresponding insulin areas. Thus, GI and II were calculated using the net incremental AUC (0-120 min), with WWB as a reference. Incremental peaks for glucose and insulin were calculated as maximum postprandial increase from baseline.

The glycemic profile (GP) defined as the duration of the glucose curve divided with the incremental glucose peak was calculated [18], with the modification that in cases where the glucose remained above fasting for the entire 270 min before lunch, the duration value was set to 270 min.

Hydrolysis index were calculated from the 180 min AUC for in vitro starch hydrolysis, in a similar way of calculating GI and II values, using WWB as a reference [30].

Time x treatment interactions for glucose, insulin, ghrelin, satiety, breath hydrogen, FFA and adiponectin responses were analyzed using a mixed model (PROC MIXED in SAS release 8.01, SAS Institute Inc., Cary, NC) with repeated measures and an autoregressive covariance structure. Subjects were modeled as a random variable and corresponding baseline (fasting values) value were modeled as covariate. The data were analyzed using a mixed model analysis of covariance (ANCOVA) with subject as a random variable and corresponding baseline (fasting values) as a covariate. For voluntary energy intake at lunch and HI, a mixed model analysis of variance (ANOVA) was used with subject as a random variable. Differences between groups were identified using Tukey's multiple comparison tests. (MINITAB, release 16, Minitab Inc., State College, PA). In the cases of unevenly distributed residuals (tested with Anderson-Darling test), Box Cox transformation were performed on the data prior to the ANCOVA and ANOVA. Correlation analysis was conducted to evaluate the relation among dependent measures with the use of Spearman's partial correlation coefficients controlling for subjects and corresponding baseline values (two-tailed test), (SPSS software, version 19; SPSS Inc., Chicago, IL, USA). p < 0.05 was considered statistically significant.

## Results

### Starch hydrolysis (HI)

The rye products and WK displayed a lower rate of starch hydrolysis, expressed as HI, than did WWB (Table 1). Furthermore, RK and WK displayed lower HI's than the rye breads.

### Glucose responses following breakfast

**Figure 1:**
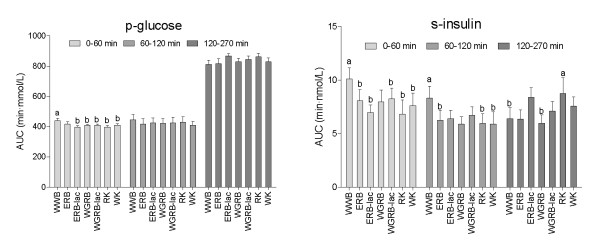
**Glucose and insulin responses after the breakfast products**. Values are means ± SEM, n = 10 (n = 9 for WGRB). Products not sharing the same letters were significantly different. Products not displaying letters were not different from any other test product, p < 0.05 (ANCOVA, followed by Tukey's test).

All products except the ERB displayed lower glycemic indices (GI's) than WWB, with GI's ranging from 64 to 79 (Table 2). WK and all rye products, except ERB, induced lower early glucose responses than WWB (AUC 0-60 min, Figure 1). The incremental glucose peak was lowered following all products in comparison to WWB, with the exceptions of ERB. The glycemic profile (GP, min·mmol^-1·^L^-1^) was higher for RK than for WWB and WK. Generally, rye products induced higher GP's than wheat products (mean GP 74 for rye and 50 for wheat) No time x treatment interaction was found (0-270 min).

**Table 2:** Glucose and insulin responses and after the breakfast products

Meals	GI	Incremental glucose peak	GP	Incremental insulin peak	II
	***%***	***Δ mmol/L***	***min·mmol^-1·^L^-1·^***	***Δ nmol/L***	***%***
WWB	100^a^	3.9 ± 0.4 ^a^	49 ± 7 ^b^	0.250 ± 0.029 ^a^	100 ^a^
ERB	77 ± 8 ^ab^	3.2 ± 0.3 ^ab^	59 ± 10 ^ab^	0.177 ± 0.026 ^b^	68 ± 4 ^b^
ERB-Lac	64 ± 9 ^b^	2.5 ± 0.3 ^b^	78 ± 9 ^ab^	0.152 ± 0.017 ^b^	65 ± 7 ^b^
WGRB	79 ± 14 ^b^	2.7 ± 0.2 ^b^	75 ± 13 ^ab^	0.180 ± 0.026 ^b^	70 ± 5 ^b^
WGRB-Lac	64 ± 7 ^b^	2.6 ± 0.2 ^b^	65 ± 9 ^ab^	0.180 ± 0.025 ^b^	75 ± 8 ^b^
RK	73 ± 8 ^b^	2.5 ± 0.3 ^b^	94 ± 13 ^a^	0.140 ± 0.027 ^b^	60 ± 7 ^b^
WK	68 ± 9 ^b^	3.0 ± 0.4 ^b^	51 ± 7 ^b^	0.173 ± 0.029 ^b^	63 ± 9 ^b^

Values are means ± SEM, n = 10 (n = 9 for WGRB). Products not sharing the same letter are significantly different, p < 0.05 (ANCOVA, followed by Tukey's test).

### Insulin responses following breakfast

All breakfast products induced lower insulinemic indices than WWB ranging from 60 for the kernel based products to 75 for the whole grain rye bread (Table 2). In the early postprandial phase all products except WGRB induced lower insulin responses than WWB (AUC 0-60 min, Figure 1). All products induced lower incremental insulin peak values than WWB. No time x treatment interaction was found (0-270 min)

### H_2 _excretion following breakfast and buffet lunch

**Figure 2:**
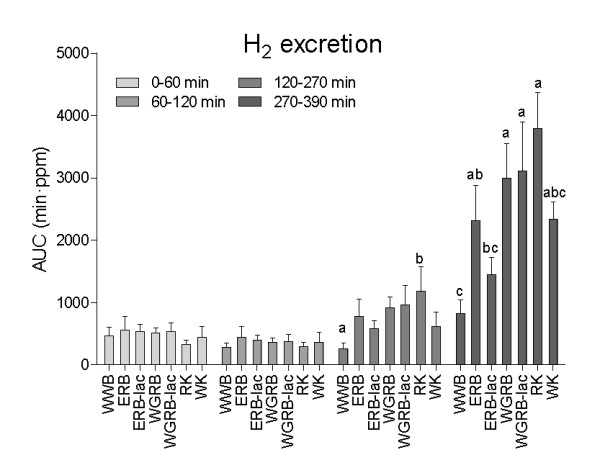
**H2 excretion after the breakfast products**. Values are means ± SEM, n = 10 (n = 9 for WGRB). Products not sharing the same letters were significantly different. Products not displaying letters were not different from any other test product, p < 0.05 (ANCOVA, followed by Tukey's test).

Prior to lunch, RK induced higher H_2 _excretion than WWB (AUC 120-270 min, Figure 2). All products except ERB-lac and WK induced higher increment in H_2 _than WWB in the postprandial phase following the buffet lunch (AUC 270-390 min). Furthermore, RK, WGRB and WGRB-lac generated higher H_2 _than ERB-lac following the buffet lunch (AUC 270-390 min)

Significant differences in H_2 _were observed at specific time points (time x treatment p = 0.0016 for the 0-270 min interval). At 240 min, WGRB induced higher H_2 _excretion than WWB and ERB and at 270 min; RK and WGRB induced higher H_2 _excretion than WWB and ERB-lac. No significant time x treatment interaction was found for the interval 270-390 min.

### Subjective appetite ratings following breakfast and buffet lunch

**Figure 3:**
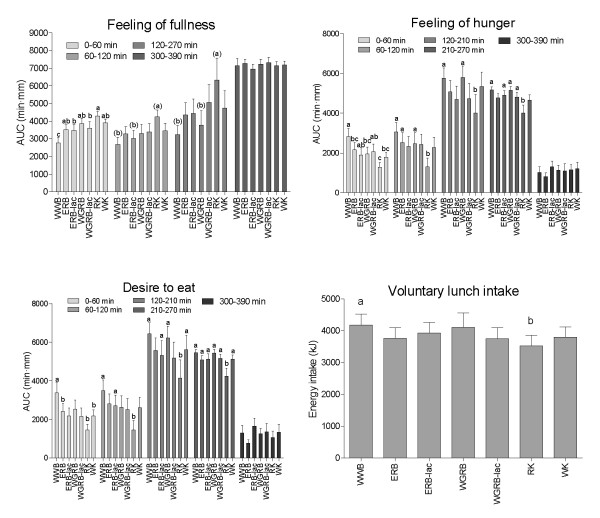
**Subjective satiety and voluntary energy intake at lunch after the cereal test breakfasts**. Values are means ± SEM, n = 10 (n = 9 for WGRB). Products not sharing the same letters were significantly different. Products not displaying letters were not different from any other test product, p < 0.05 (ANCOVA, followed by Tukey's test). ( ) indicates that the ANCOVA did not have normal distributed residuals, p < 0.05. Voluntary lunch intake was analyzed with ANOVA followed by Tukey's test

All test products induced a higher *feeling of fullness *and all but ERB-Lac, WGRB-lac and WGRB induced less *desire to eat *than WWB in the early postprandial phase after breakfast (AUC 0-60 min, Figure 3). During this phase, the RK breakfast also induced higher *feeling of fullness *than did WGRB-lac and ERB-lac.

Feeling of hunger was higher following the WWB compared to after RK, WK, ERB and WGRB in the early postprandial phase (0-60 min). Furthermore, WGRB-lac and ERB-lac induced higher feeling of hunger than RK breakfast in this interval. In the later postprandial phase (120-210 min), the test subjects felt hungrier and had a larger desire to eat following the WWB and WGRB breakfasts compared to following the RK breakfast. Furthermore, ERB-lac and WK caused a larger desire to eat compared to RK in this phase. In the hour prior to lunch (210-270 min), hunger was higher following all test products but ERB and WK compared to after the RK breakfast while desire to eat was higher for all breakfast, compared to after RK.

There were no differences in *feeling of fullness, hunger *or *desire to eat *after the ad lib lunch following the breakfast meals (AUC 300-390), indicating that subjects succeeded in eating to the same degree of satiation, as was intended in the experimental design. No time x treatment effect was found for *feeling of fullness, feeling of hunger *or *desire to eat *in the intervals 0-270 or 300-390.

### Voluntary lunch intake

To reach the same degree of satiation, subjects decreased their energy intake (EI) at lunch after the RK breakfast compared with the corresponding intake after the WWB breakfast with 16% (Figure 3). Also the cumulative EI over the breakfast- and lunch meals was lower on the RK breakfast test day, compared to the WWB breakfast test day.

### Ghrelin responses following breakfast and buffet lunch

**Figure 4:**
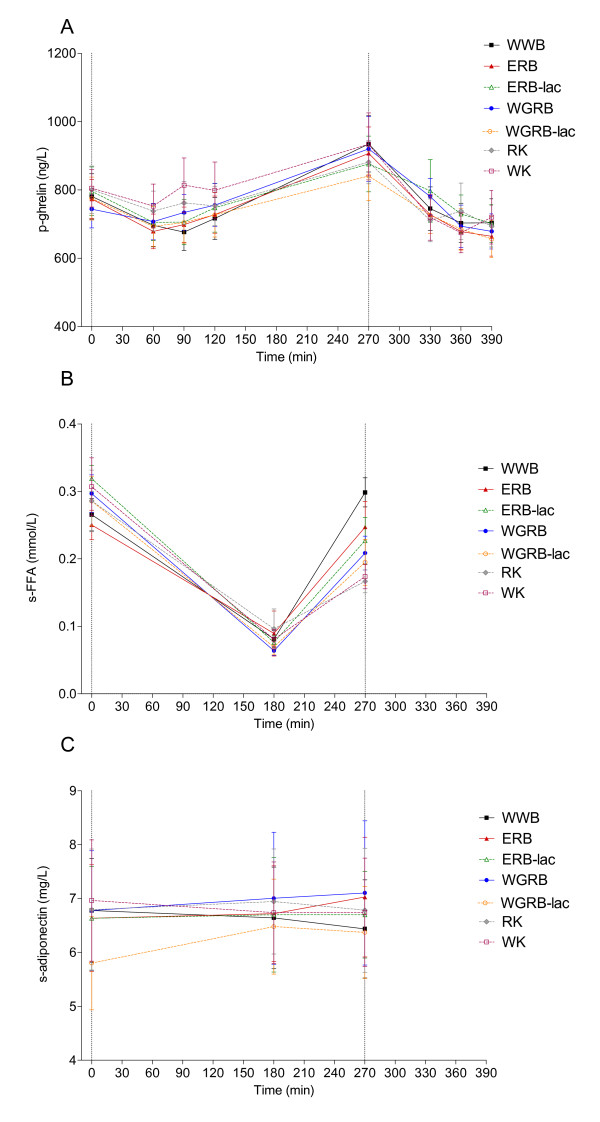
**Postprandial change in ghrelin, (A), FFA (B) and adiponectin (C)**. Values are means ± SEM, n = 10 (n = 9 for WGRB).

For ghrelin, WGRB-lac induced lower ghrelin AUC in the postprandial period 120-270 min compared to WK. No differences were found between AUC following the different products, in the interval 270-390 min. No time x treatment interaction was found in the interval 0-270 or 270-390 min. (Figure 4).

### Free fatty acids

RK induced lower concentrations of FFA than WWB in the later postprandial phase (AUC 180-270 min, Figure 4). A time x treatment interaction was found in the interval 0-270 min (p < 0.001) and the WWB induced higher FFA at 270 min compared to WK and RK, respectively.

### Adiponectin following breakfast

For adiponectin (0-270 min), no change over time, and no time x treatment interaction was found. Nor was there any difference in AUC 0-270 min or 180-270 min between the products (Figure 4).

### Correlations between parameters

Correlations between appetite ratings, physiological parameters and product properties are presented in **Tables **3 and 4. A low II, incremental insulin peak, and GI, respectively, was related to a higher *feeling of fullness, lower feeling of hunger *and lower *desire to eat *the first 60 min after breakfast. A high GP was related to a lower *desire to eat*, both in the early and *late *postprandial phase (0-60 and 210-270 min). Low late desire to eat (AUC 210-270 min) was also related to a lower EI at the subsequent lunch. A high GP was related to a lower concentration of ghrelin prior to lunch (270 min), in turn correlated with a lower EI. Furthermore, a higher content of indigestible carbohydrates, as well as high water content and larger portion sizes correlated with increased *feeling of fullness *and a lowered *feeling of hunger *and *desire to eat (0-60 min) *as well as lowered EI at lunch. High content of indigestible carbohydrates correlated with a high H_2 _excretion (120-270 min), which, in turn, was related to a lower EI at lunch. A high H_2 _excretion (90-270 min) correlated to a higher GP. GP and GI did not correlate. HI for the rye breads was not related to GI, II or GP.

**Table 3:** Correlations between appetite ratings, physiological parameters and product properties

	*feeling of fullness *(AUC 0-60 min)	*feeling of hunger *(AUC 0-60 min)	*desire to eat *(AUC 0-60 min)	*desire to eat *(AUC 210-270 min)	Energy intake at lunch (kJ)
	***r***				

*Desire to eat *(AUC 210-270 min)	-0.17, p = 0.18	0.11, p = 0.39	0.21, p = 0.095		**0.43, p < 0.001**

Energy intake at lunch (kJ)	**-0.25, p = 0.042**	0.18, p = 0.15	**0.31, p = 0.013**	**0.43, p < 0.001**	

II	**-0.36, p = 0.004**	**0.37, p = 0.003**	**0.42, p = 0.001**	0.17, p = 0.18	**0.30, p = 0.013**

Incremental insulin peak	**-0.38, p = 0.002**	**0.36, p = 0.003**	**0.44, p < 0.001**	0.22, p = 0.082	**0.40, p = 0.001**

GI	**-0.48, p < 0.001**	**0.44, p < 0.001**	**0.39, p = 0.002**	0.04, p = 0.74	0.02, p = 0.87

GP	0.25, p = 0.050	-0.23, p = 0.067	**-0.30, p = 0.016**	**-0.29, p = 0.020**	-0.22, p = 0.078

Indigestible carbohydrate (g)	**0.68, p < 0.001**	**-0.48, p < 0.001**	**-0.56, p < 0.001**	**-0.25, p = 0.045**	**-0.34, p = 0.005**

Water content (g)	**0.65, p < 0.001**	**-0.48, p < 0.001**	**-0.55, p < 0.001**	**-0.29, p = 0.017**	**-0.36, p0.002**

Portion size (g)	**0.65, p < 0.001**	**-0.48, p < 0.001**	**-0.55, p < 0.001**	**-0.29, p = 0.017**	**-0.36, p = 0.002**

H_2 _(AUC 120-270 min)				-0.24, p = 0.053	**-0.34, p = 0.005**

H_2 _(AUC 270-390 min)					**-0.33, p = 0.007**

Ghrelin (270 min)				-0.04, p = 0.77	**0.33, p = 0.006**

Ingestible carbohydrate content consists of total fiber + resistant starch in breakfast products. Spearman's partial correlation coefficients controlling for subjects and corresponding baseline values (two-tailed test). Significant correlations are shown in bold text.

**Table 4:** Correlations between physiological parameters and certain product properties

	r	p
GP vs. II	**-0.39**	**0.001**
GP vs. Incremental insulin peak	**-0.43**	**>0.001**
GP vs. GI	-0.22	0.072
GP vs. Ghrelin (270 min)	**-0.29**	**0.018**
Indigestible carbohydrate (g). vs. H_2 _(AUC 120-270) min	**0.46**	**>0.001**
Indigestible carbohydrate (g). vs. H_2 _(AUC 270-390 min	**0.70**	**>0.001**
GP vs. H_2 _(AUC 90-270) min	**0.29**	**0.018**
GP vs. HI (rye breads)	-0.11	0.50
GI vs. HI (rye breads)	0.121	0.48
II vs. HI (rye breads)	-0.10	0.56

Ingestible carbohydrate content consists of total fiber + resistant starch in breakfast products. Spearman's partial correlation coefficients controlling for subjects and corresponding baseline values (two-tailed test). Significant correlations are shown in bold text.

## Discussion

In the present study, we confirm previous findings of a low insulin response and a high Glycemic Profile following rye products. Furthermore, we demonstrate that rye products beneficially affect both early and late appetite regulation, making them an interesting food component in weight management. The RK breakfast, in particular, improved appetite regulation and increased satiety acutely and at a subsequent meal, as judged from a lower energy intake. Consequently, compared with WWB, RK induced improved satiety ratings in the early postprandial phase after breakfast, and substantially lowered the voluntary energy intake (-16%) at a subsequent lunch. As a result of this the cumulative energy intake (breakfast + lunch) was lower on the RK day compared to the WWB test day.

Part of the early satiating effect of RK may be explained by portion size. Despite being similar to the other test products regarding energy content, the RK breakfast had the largest volume of the test products. The difference in size of the test products can be explained by the fact that RK was the test product containing the highest amount of indigestible carbohydrates (dietary fiber + resistant starch), contributing to a higher water-binding capacity during processing (cooking). Improved early (0-60 min) satiety ratings after the test meals correlated well with a high content of ingestible carbohydrates, a large portion size and a high water content in the products. The satiating effect of bulk-inducing indigestible carbohydrates and water has previously been demonstrated by Rolls et al. [32] who showed that the amount of food eaten, but not the energy content of the foods affect satiety in healthy men. Also, Geliebter demonstrated in 1988 [33] that larger distension of the stomach reduced voluntary food intake in both lean and obese subjects.

A low voluntary energy intake at lunch could be explained by increased colonic fermentation; Breath H_2 _has been shown to be a sensitive indicator of increased carbohydrate fermentation in colon [34] and a high breath H_2 _prior to lunch (120-270 min) was related to a following lowered voluntary energy. Rye products tended to increase breath H_2 _prior to lunch, significantly so following the RK and WGRB breakfasts. After lunch, all rye products except the ERB-lac induced higher H_2 _excretions than WWB. All rye products had a high content of soluble fibers, probably explaining the early fermentation. Colonic fermentation of indigestible carbohydrates yields SCFA [35], which may promote feeling of satiety through a relaxation of the gastric tone and a slower gastric motility [36]. In support of the fermentation hypothesis; Cani et al [37] has demonstrated lowered energy intake, increased GLP-1 secretion and lowered ghrelin secretion in rats fed a high fructan diet. Furthermore, Nilsson et al. [38] demonstrated that an evening meal consisting of bread made from barley kernels, rich in dietary fiber and resistant starch, increased subjective satiety and reduced gastric emptying rate at a subsequent standardized breakfast in healthy subjects. The benefits on satiety were assigned to colonic fermentation and stimulation of GLP-1. Taken together, these findings demonstrate the potential of colonic fermentation as a modulator of satiety.

Improved satiety ratings in the early postprandial phase after breakfast and a lowered voluntary energy intake at lunch were also associated with a low insulin response (II and incremental insulin peak) following the breakfast test meals. All rye products and the WK were characterized by a lower II than WWB. In a review by de Graaf et al. [39], insulin was suggested to be a poor biomarker of satiety, since it is confounded or moderated by several metabolic processes such as blood glucose and incretins. However, the current finding that a low postprandial insulin response correlates with improved satiety ratings and a lowered energy intake at a following meal is supported by several studies [18,21-25,40,41], with equi-carbohydrate portions and similar macronutrient compositions in the test foods; that is, at conditions comparable to those in the present study. Possibly, it is the absorption characteristics of the carbohydrates rather than insulin concentrations *per se *that affect satiety. A slow uptake of carbohydrates would lead to a prolonged exposure of the small intestine to nutrients, thus extending the release of satiety peptides, e.g. GLP-1 [42-44]. Intake of food products containing slowly absorbable carbohydrates will require smaller amounts of insulin for the glucose uptake, thus limiting the risk of reactive hypoglycemia in the later postprandial phase. We have recently demonstrated that a product characterized by a low but prolonged blood glucose curve, described by a high Glycemic Profile (GP) was associated with lower insulin response, less postprandial hypoglycemia, and a smaller increase in late postprandial ghrelin [18]. In the present work, a high GP after a meal appears to positively affect appetite regulation, partly by reduced secretion of ghrelin. A high GP was associated with both a low postprandial concentration of ghrelin at 270 min, and a lower *desire to eat *both in the early and late postprandial phase (AUC 0-60 and 210-270 min). Furthermore, a lower concentration of ghrelin and a lower *Desire to eat *was related to a lower voluntary energy intake.

Rye products were characterized by higher GP than wheat products. Looking at specific rye products, RK induced significantly higher GP than WWB and WK. This is in line with our previous findings [18]. In the present work, the GP of the products were inversely related to the insulin response (II and incremental peak) but not to the GI. A prolonged blood glucose curve seen with high GP rye products could, besides a lowered insulin response, be a result of improved glucose tolerance induced by colonic fermentation [45]. Colonic fermentation has recently been demonstrated to increase peripheral insulin sensitivity, 10 h after the test meal [46]. In the present study, a high breath hydrogen excretion was detected already in the 90-270 min phase after the test breakfast, and was found to correlate to a high GP. Additionally, the lowered blood glucose incremental peak seen with the high GP rye products could be explained by bioactive components, and by a lowered starch hydrolysis. Although no direct correlations between the GP, GI or II of the rye breads and the rate of in vitro starch hydrolysis (HI) could be found, the HI was lowered for the rye products compared to WWB. Obstructed amylolysis could therefore partly contribute to the low glucose incremental peaks and insulin responses of most rye products in the study. That the GP of RK was higher than that of WK suggests a facilitated glucose regulation following the RK breakfast. Interestingly, RK and WK induced similar GI values which indicate the importance of GP as a complement to GI, where the latter does not provide information about the course of glycemia.

## Conclusions

Rye products, especially in the form of whole kernels, decrease both early and late appetite ratings after a breakfast meal, and lowers energy intake at a subsequent voluntary lunch. Our results suggest that a high content of indigestible carbohydrates and soluble fibers in the rye products may beneficially affect acute satiety through a bulking effect, and second meal satiety through a mechanism related to colonic fermentation by production of fermentation metabolites. Colonic fermentation might also contribute to an improved late glucose regulation. Indications of such a hypothesis stems from the correlation between a high breath hydrogen excretion and a high GP. A high GP was also related to lowered insulin response, late postprandial ghrelin secretion and desire to eat, thereby affecting a subsequent *ad libitum *meal. These findings indicate that the GP represent a nutritionally interesting entity in that it predicts metabolic responses as well as the satiating properties better than does the GI. This work provides information of a potential role in weight management for rye products. A high intake of rye products could contribute to a lowered energy intake, and thus protect against obesity. To evaluate this hypothesis, longer-term studies of rye products on metabolism and appetite regulation are needed.

## List of abbreviations

AUC: total area under the curve; BMI, body mass index; CVD: cardiovascular diseases; EI: voluntary energy intake at lunch; ERB-lac: endosperm rye bread made with lactic acid; ERB: endosperm rye bread; FFA: free fatty acids; GI: glycemic index; GP: Glycemic profile; H_2, _breath hydrogen; HI: hydrolysis index; II: insulinemic index; RK: Rye kernels; T2D: type 2 diabetes; WGRB: whole grain rye bread; WGRB-lac: whole grain rye bread made with lactic acid; WK: wheat kernels; WWB: white (endosperm) wheat bread.

## Competing interests

The authors declare that they have no competing interests.

## Authors' contributions

LAHR coordinated the study and was responsible for the study design, the collection and analysis of the data, statistical analysis and for writing the paper. EMÖ was involved in the study design, interpretation of data and in writing the paper. IMEB was the guarantor and was involved in the study design, interpretation of data and writing of the paper. All authors have read and approved the final manuscript.
